# Cytomegalovirus (CMV) Viremia Presenting as a Heart Failure Exacerbation

**DOI:** 10.7759/cureus.108181

**Published:** 2026-05-03

**Authors:** Chirag Lodha, Eric J Basile, Suhaas Singireddy

**Affiliations:** 1 Internal Medicine, University of South Florida Morsani College of Medicine, Tampa, USA; 2 Cardiovascular Disease, University of South Florida Morsani College of Medicine, Tampa, USA

**Keywords:** cardiology, cmv viremia, heart failure, heart failure with reduced ejection fraction, infectious disease, kidney transplant, transplant

## Abstract

A 68-year-old male renal transplant recipient with a past medical history including chronic kidney disease (CKD) stage 3A due to type 2 diabetes mellitus, renal cell carcinoma, atrial fibrillation, prior stroke, prostate cancer post-prostatectomy, and heart failure with preserved ejection fraction (HFpEF) presented to the emergency department with worsening shortness of breath. Vital signs showed B-type natriuretic peptide (BNP) at 1,076 pg/mL, troponin at 55 ng/L without ischemic ECG changes, creatinine at 1.2 mg/dL below his baseline of 1.6 mg/dL, and a subtherapeutic international normalized ratio (INR) of 1.54. Chest X-ray revealed mild fluid congestion, leading to a diagnosis of heart failure exacerbation. Transplant nephrology consultation prompted tacrolimus level measurement, revealing supratherapeutic levels at 15.5 ng/mL, resulting in its hold. Concern for immunosuppression due to supratherapeutic tacrolimus levels prompted cytomegalovirus (CMV) and BK virus testing; BK was negative, but CMV viral load reached 106,297 IU/mL, with a positive viral load indicating active infection. Mycophenolate was held, and the patient developed a fever to 102.6°F before starting treatment for CMV viremia that resolved spontaneously.

Oral valganciclovir 450 mg every 12 hours initiated CMV treatment. Renal ultrasound showed no hydronephrosis or rejection in native or transplant kidneys. He was discharged to a skilled nursing facility, with monitoring until his viral load cleared, followed by three additional months of additional valganciclovir therapy.

## Introduction

Cytomegalovirus (CMV) infection poses a major threat to adult kidney transplant recipients due to immunosuppressive therapy that impairs viral control [1]. Viremia occurs when the virus replicates in the bloodstream and often leads to tissue-invasive disease without intervention. PCR assays detect CMV DNA and guide management in these patients. Incidence rates vary widely but reach approximately 22-40% in high-risk groups without prophylaxis [2]. Risk stratification based on serostatus directs the choice between universal prophylaxis and preemptive therapy. Early post-transplant months see peak viremia episodes, particularly in seronegative recipients. Longitudinal monitoring reveals patterns of primary infection and reactivation. National registries track these trends and inform protocol updates [3]. Seroprevalence exceeds 50% in many adult populations, which influences donor-recipient matching. D+/R- mismatches carry infection rates up to 70% without prophylaxis, though regimens reduce this to 15-30% [4].

Direct effects damage graft endothelium, while indirect effects heighten rejection and opportunistic infections. CMV viremia can present in a variety of ways, such as recurrent fevers, sepsis, as well as in other atypical ways, such as volume overload or shortness of breath. This presentation is also highly variable depending on the solid organ transplant; however, it is atypical for CMV viremia, causing shortness of breath and volume overload in the case of kidney transplant patients. This underlying insult can disrupt the delicate homeostasis of transplant patients and can be the underlying insult to patient symptomatology when they have otherwise been doing well after their solid organ transplant.

Epidemiology

CMV viremia peaks between one and six months post-transplant in at-risk kidney recipients. Without prophylaxis, about 60% of high-risk patients develop viremia [5-7]. D+/R- pairs experience rates as high as 70%, with universal prophylaxis shifting risks to late-onset disease [4]. Studies confirm five-year analyses show persistent threats despite interventions [2]. Geographic factors affect donor seroprevalence, with higher rates in urban areas. Donor age influences outcomes; viremia harms grafts more in recipients of donors under 70 years, likely due to higher antibody titers in these patients upon donating their organs [3]. Registries like OPTN provide incidence benchmarks and track reductions from modern protocols [7]. Patterns include early primary infections in D+/R- cases and reactivation in seropositive recipients. Delayed-onset viremia emerges after prophylaxis ends [5]. Surveillance over 12 months captures these dynamics, with high loads signaling progression.

Breakthrough infections occur despite prophylaxis, especially with lower-dose valganciclovir in D+/R- recipients [1]. Pediatric and adult recipients show similar viremia rates, though side effects differ [7]. Coinfections with Epstein-Barr virus (EBV) or BK virus increase readmissions during outbreaks. National data highlight 20% incidence drop from protocol changes [7]. Longitudinal studies in large cohorts with extended criteria donors affirm viremia's role in early graft threats [3]. QuantiFERON-CMV assays fail to predict viremia reliably in seropositive recipients from high-seroprevalence areas [8]. Torque teno virus (TTV) load combined with immune assays better stratifies reactivation risk in R+ patients [9]. These epidemiological insights guide center-specific strategies.

Risk factors

Donor-recipient serostatus drives CMV risk, with D+/R- pairs at highest danger for viremia and disease [1,10]. T-cell depleting induction, like antithymocyte globulin, triples viremia odds [11]. Older recipients and those with diabetes face added susceptibility from immune decline [3]. Human leukocyte antigen (HLA) mismatches and re-transplantation contribute indirectly. Mechanical ventilation and intense immunosuppression regimens elevate threats [2]. Genetic factors affecting CMV receptors may modulate severity [7]. Pretransplant screening and validated tools inform prophylaxis duration. Tacrolimus-based therapy is linked to lower viremia than cyclosporine [10]. Donor age under 70 years amplifies viremia's graft loss impact in R+ recipients [3]. Coinfections like BK alter dynamics, potentially associated with protection against CMV, as a pause in the immunosuppression regimen leads to improved immunological response against these pathogens [6].

Pathophysiology involves viral entry via monocytes, latency in salivary glands and bone marrow, and reactivation under immunosuppression [2]. Endothelial invasion causes glomerulopathy and accelerates atherosclerosis [10]. High viral loads correlate with injury. Alemtuzumab induction with low-dose valganciclovir reduces incidence but raises rejection [11]. Risk assessment integrates serology, induction type, and age. Studies urge more research on prophylaxis to curb early infection and rejection [10]. QuantiFERON-CMV assays guide tailored prophylaxis in R+ but not D+/R- patients [12]. TTV viremia predicts reactivation when paired with QuantiFERON metrics [9]. These factors shape individualized prevention.

## Case presentation

A 68-year-old male with a past medical history of chronic kidney disease (CKD) stage 3A secondary to type two diabetes mellitus, renal cell carcinoma on mycophenolate, tacrolimus, and dapsone, atrial fibrillation on warfarin, stroke without residual deficits, prostate cancer status post-prostatectomy, and heart failure presented to the ED for worsening shortness of breath. In the ED, his vitals were remarkable for a B-type natriuretic peptide (BNP) that was elevated, a mildly elevated troponin without any acute ischemic changes on ECG, and a creatinine of 1.2, decreased from his baseline of approximately 1.6 (Table 1).

**Table 1:** Pertinent lab values

Lab	Patient Value	Normal Value
B-type natriuretic peptide (pg/mL)	1076	<100
Troponin (ng/L)	55	<50
Creatinine (mg/dL)	1.2	<1.1
International normalized ratio	1.54	0.8-1.1

**Figure 1:**
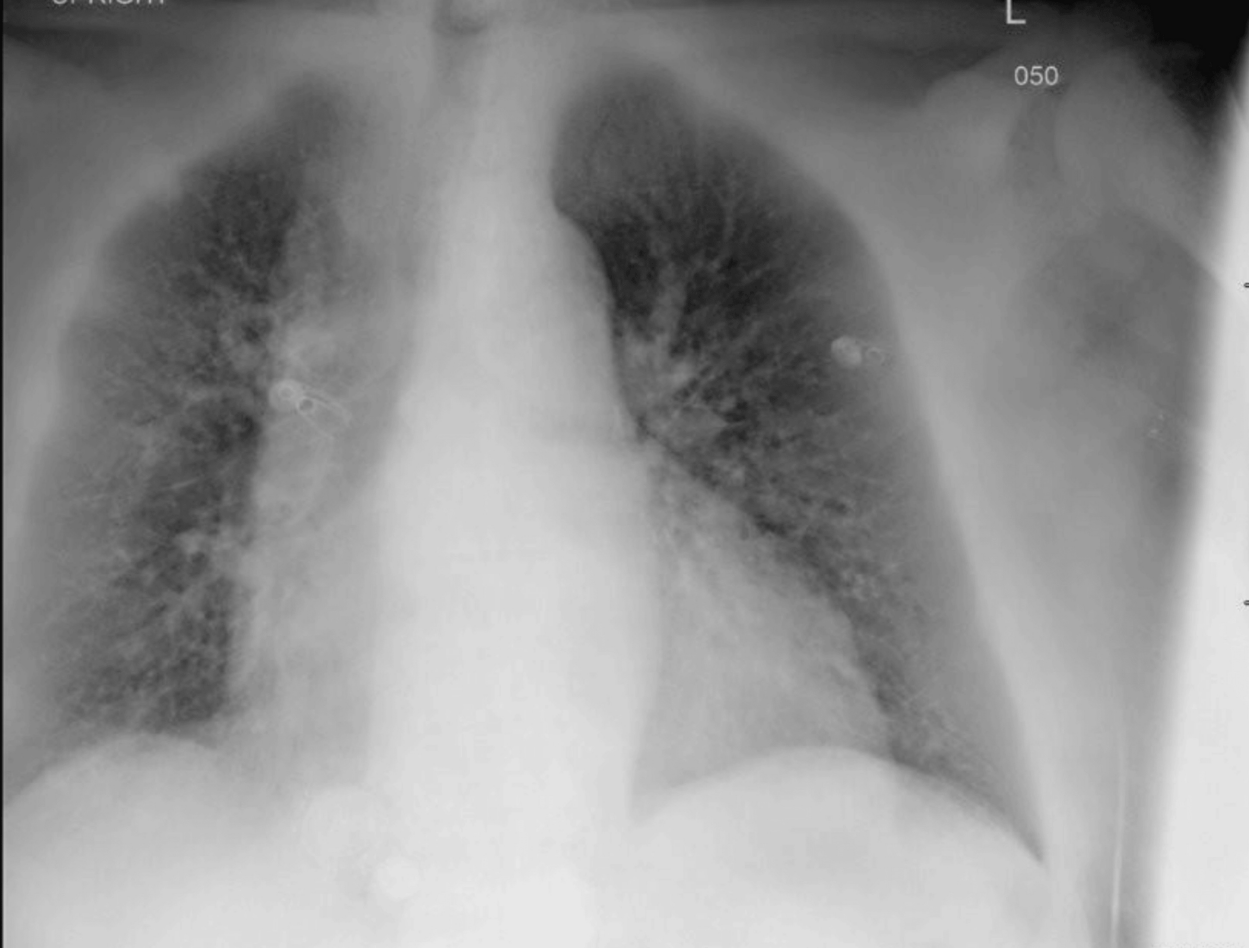
Chest X-ray showing pulmonary vascular congestion

His international normalized ratio (INR) was subtherapeutic at 1.54 (Table 1). At this point, the differential included heart failure exacerbation due to the shortness of breath and history of heart failure with reduced ejection fraction (HFrEF), or acute rejection of his transplanted kidney, possibly due to infection or his supratherapeutic tacrolimus level. Therefore, a chest X-ray was ordered, which showed mild fluid congestion, and he was diagnosed with a heart failure exacerbation (Figure 1). This was due to his clinically overloaded volume status as well as elevated BNP, with imaging findings corroborating these findings (Figure 1).

**Figure 2:**
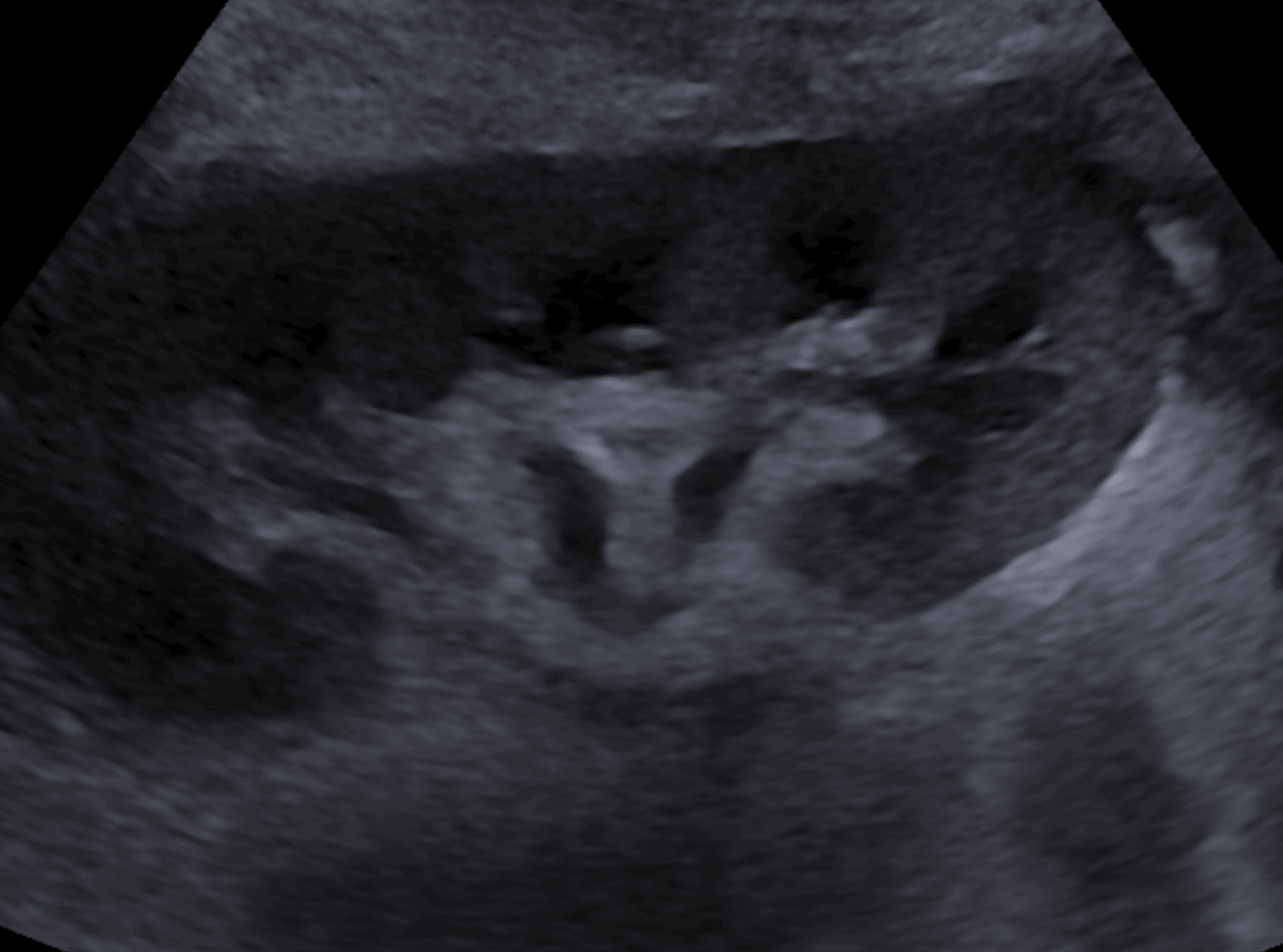
Transplant kidney ultrasound showing no evidence of hydronephrosis

He received 40 mg IV furosemide with good urine output. Due to the history of a renal transplant, transplant nephrology was consulted, and a tacrolimus level was drawn, found to be significantly elevated at 15.5 ng/mL (Table 1), which was subsequently held. Due to his supratherapeutic tacrolimus level, there was concern that the patient may have been too immunocompromised, and therefore, CMV and BK virus serology were drawn due to concern that an infection may have placed him into a heart failure exacerbation. He was negative for the BK virus, but his CMV viral load resulted in 106,297 IU/mL on day 3 of his admission. His mycophenolate was then held due to CMV viremia, and he spiked a temperature of 102.6°F the same day, which improved spontaneously without any anti-pyretics. He was started on PO (by mouth) valganciclovir 450 mg PO every 12 hours, due to his mildly increased creatinine, raising concern for compromised renal function, as well as the nephrotoxicity associated with supratherapeutic levels of tacrolimus. He was briefly switched to IV due to a fever of 101.7°F, but due to the resolution of the fever, he was switched back to valganciclovir after four days. His hospital stay was complicated by numerous supratherapeutic tacrolimus levels despite decreasing his dosage, as well as a labile INR, attributed to poor PO intake. There was concern for renal failure due to a brief increase in lower extremity edema while having a tacrolimus level of 11 ng/mL, for which he was diuresed with 1 mg IV Bumex, resulting in an acute kidney injury (AKI) secondary to diuretic use, with a creatinine peaking at 3.3 mg/dL, which resolved after gentle IV normal saline hydration over three days. The renal ultrasound of his functional native kidney and transplanted kidney showed no hydronephrosis or acute concerns of rejection (Figure 2).

Urology evaluated the patient and stated that he may have a urethral stricture and would benefit from a cystoscopy as an outpatient. Once his INR and tacrolimus levels normalized, he was discharged to a skilled nursing facility (SNF) due to chronic deconditioning with instructions to continue PO valganciclovir 450 mg with serial CMV viral load draws. Once his viral load became negative, he continued the aforementioned treatment for a total of three additional months.

## Discussion

We present a case of CMV viremia presenting atypically as a heart failure exacerbation. Although it was believed to be an isolated incidence of heart failure exacerbation, suspicion was raised due to his supratherapeutic tacrolimus level, leading to providers obtaining a CMV viral load. This clinical course was complicated by worsening renal function due to a tenuous volume status, which ultimately improved with intravenous fluid resuscitation. He was placed on antiviral therapy with resolution of fevers while waiting for infectious disease follow-up as an outpatient to draw further CMV viral loads. 

CMV viremia remains a major cause of transplant organ rejection [2]. It accelerates chronic nephropathy and proteinuria in 40% of cases in some observational studies [3,4]. Hospital stays extend by 10 days per episode in some cohorts, with bacterial superinfections in 30% [6]. Healthcare costs rise 50% in transplanted patients, and quality of life significantly declines [5]. Indirect effects include vascular events like myocardial infarction and worsened glycemic control in diabetics. BK nephropathy co-occurs in 15% of transplant patients [6]. Five-year graft survival drops from 85 to 65% [13]. Meta-analyses report 1.8 hazard ratios for dysfunction. Malignancy risks grow with prolonged viremia. Multidisciplinary care mitigates some impacts. 

Symptomatic disease features include fever in 80%, leukopenia in 40-60%, and thrombocytopenia in 30% of solid organ transplant patients [14]. Gastrointestinal symptoms like diarrhea affect many, with symptoms such as retinitis or pneumonitis rarely. Allograft dysfunction signals renal involvement, confirmed by inclusions in 25% of biopsies [15]. Therefore, when treating patients with a history of solid organ transplant and acute decompensation, providers should remain vigilant to obtain a medication level for their immunosuppressant. If supratherapeutic, clinicians should have a high index of suspicion to obtain a CMV viral load. Delayed diagnosis worsens outcomes, with PCR tracking progression of resolution [1]. De novo infection can manifest as interstitial nephritis with urologic issues [15]. High-volume centers report better control, possibly due to increased familiarity with managing CMV infection [14]. 

Untreated CMV viremia carries 20-50% mortality, with viremia alone raising one-year death risk 2.5-fold [6]. Resistant strains double the risks of complications, such as organ rejection [16]. Registry data show a 5-10% overall impact in solid organ transplant patients, reduced to 5% with therapy [8]. Renally adjusted dosing (450 mg) matches standard-dose efficacy with decreased incidence of leukopenia in those undergoing prophylactic treatment of CMV with renal impairment [17,18]. Alternate-day dosing works in moderate-risk D+/R+ cases [19,20].

## Conclusions

In conclusion, CMV viremia accounts for a significant amount of morbidity and mortality in transplant patients, especially within the first year. Caution must be exercised when treating transplant patients, and ascertaining CMV donor status is critical in the management of these patients. Regardless of donor status, clinicians should be aware of CMV infection as a cause of clinical decompensation and atypical presentations of solid organ transplant recipients, such as volume overload and shortness of breath.
